# Draft Genome Sequence of *Bacillus* sp. Strain K2I17, Isolated from the Rhizosphere of *Deschampsia antarctica* Desv.

**DOI:** 10.1128/genomeA.00786-17

**Published:** 2017-08-17

**Authors:** Alequis Pavón, Paz Orellana, Lorena Salazar, Sandra Céspedes, Laura Muiño, Ana Gutiérrez, Daniel Castillo, Gino Corsini

**Affiliations:** a Facultad de Ciencias de la Salud, Instituto de Ciencias Biomédicas, Universidad Autónoma de Chile, Santiago, Chile; b BIOREN-UFRO and Departamento de Producción Agropecuaria, Facultad de Ciencias Agropecuarias y Forestales, Universidad de la Frontera, Temuco, Chile; c Marine Biological Section, University of Copenhagen, Helsingør, Denmark

## Abstract

We present here the draft genome sequence of *Bacillus* sp. strain K2I17, which was isolated from the rhizosphere of *Deschampsia antarctica* Desv. The genomic sequence contained 6,113,341 bp. This genome provides insights into the possible new biomedical and biotechnical applications of this specific Antarctic bacterium.

## GENOME ANNOUNCEMENT

The genus *Bacillus* is a phenotypically large, diverse collection of Gram-positive, endospore-forming, aerobic or facultatively anaerobic bacteria that have undergone considerable reclassification due to high phylogenetic heterogeneity (1). In addition, this genus is distributed widely in extreme environments and includes psychrophilic, thermophilic, acidophilic, alkalophilic, and halophilic bacteria that utilize a wide range of carbon sources for growth (2, 3).

*Bacillus* spp. comprise one of the most interesting groups of bacteria in biotechnology due to their resistance to pH and temperature changes, which are important parameters in industrial processes (4). To date, the presence of *Bacillus* spp. has been demonstrated in different environments, including the rhizosphere, the layer of soil influenced by plant-root metabolism. In comparison to root-free soil or bulk soil, the rhizosphere displays higher contents of nutrients, which could explain the activity of *Bacillus* spp. in these dynamic environments (5), and thus new approaches have begun to utilize the biotechnological applications of *Bacillus* and related species for agricultural and pharmaceutical products (6). Here, we announce the draft genome sequence of *Bacillus* sp. strain K2I17, which was isolated from the rhizosphere of *Deschampsia antarctica* Desv., at the Collins Glacier, Fildes Peninsula, King George Island, Antarctica (62°10′S, 58°55′W).

*Bacillus* sp. strain K2I17 was grown at 18°C in LB broth (catalog no. 12106-05; Mo Bio, Inc.). Genomic DNA was extracted using the Power Biofilm DNA isolation kit (catalog no. 24000-50; Mo Bio, Inc.) according to the manufacturer’s protocol. A sequencing library was prepared using the Illumina HiSeq 2500 platform (Macrogene, Republic of Korea) with paired-end read sizes of 100 bp. A total of 15,092,729 paired-end reads were used for the *de novo* assembly in Geneious version 9.1.6 (7). Short and low-coverage contigs were filtered out, resulting in a set of 146 with an average coverage of 89× (*N*_50_, 142,492 bp). Annotation was performed with the NCBI Prokaryotic Genome Annotation Pipeline (PGAP) (8). Additionally, the genomes were analyzed on the Rapid Annotation using Subsystems Technology (RAST) server (9). Acquired antibiotic resistance genes were identified using ResFinder version 2.1 (10), virulence factors using virulencefinder version 1.2 (11), and prophage-related sequences using PHASTER (12).

The final assembly for *Bacillus* sp. strain K2I17 had a total length of 6,113,341 bp and a G+C content of 34.9%. Genome annotation resulted in 6,125 coding sequences (CDSs), 22 tRNAs, 326 pseudogenes, and 7 rRNAs; 59% of the CDSs were classified as hypothetical proteins. Five prophage-like elements between 19 and 36 kb were detected. Interestingly, one of them encodes a putative cell-adhesion protein. Genes resistant to the antibiotics tetracycline [*tet*(M) and *tet*(O)], penicillin (*bl*, *bla*, *ampS*, *pse2*, and *ybxl*), vancomycin (*vanA and vanH*), fosfomycin (*fosA*, *fosB*, and *fosX*), and fluoroquinolones (*parC* and *parE*) were detected. Also, secondary metabolism genes that encode thiazole/oxazole-modified microcin synthesis, auxin biosynthesis, pyridoxine (vitamin B_6_) biosynthesis, and lanthionine biosynthesis were identified. In addition, 27 membrane-bound and secreted *Listeria* internalin-like proteins (LPXTG and GW motifs) were identified. Thus, this genome sequence can facilitate the understanding of the genetic diversity within the *Bacillus* genus, which could be useful for the development of new biotechnological applications.

### Accession number(s).

The draft genome sequence of *Bacillus* sp. strain K2I17 has been deposited in GenBank under the accession no. NJGF00000000. The version described in this paper is the first version, NJGF01000000.

## References

[1] Logan NA, De Vos P 2015 Bacillus, p 1–164. *In* Whitman WB (ed), Bergey’s manual of systematics of archaea and bacteria, 2nd ed, vol 1 John Wiley and Sons, Hoboken, NJ.

[2] Harrell LJ, Andersen GL, Wilson KH 1995 Genetic variability of *Bacillus anthracis* and related species. J Clin Microbiol 33:1847–1850. 7665658 10.1128/jcm.33.7.1847-1850.1995 PMC228283

[3] Maughan H, Van der Auwera G 2011 *Bacillus* taxonomy in the genomic era finds phenotypes to be essential though often misleading. Infect Genet Evol 11:789–797. doi:10.1016/j.meegid.2011.02.001. 21334463

[4] Schallmey M, Singh A, Ward OP 2004 Developments in the use of *Bacillus* species for industrial production. Can J Microbiol 50:1–17. doi:10.1139/w03-076. 15052317

[5] Smalla K, Wieland G, Buchner A, Zock A, Parzy J, Kaiser S, Roskot N, Heuer H, Berg G 2001 Bulk and rhizosphere soil bacterial communities studied by denaturing gradient gel electrophoresis: plant-dependent enrichment and seasonal shifts revealed. Appl Environ Microbiol 67:4742–4751. doi:10.1128/AEM.67.10.4742-4751.2001. 11571180 PMC93227

[6] Giddings LA, Newman DJ 2015 Bioactive compounds from terrestrial extremophiles, p 1–75. *In* Tiquia-Arashiro SM, Mormile M (ed), Extremophilic bacteria, 1st ed, vol 1 Springer, New York, NY.

[7] Kearse M, Moir R, Wilson A, Stones-Havas S, Cheung M, Sturrock S, Buxton S, Cooper A, Markowitz S, Duran C, Thierer T, Ashton B, Meintjes P, Drummond A 2012 Geneious Basic: an integrated and extendable desktop software platform for the organization and analysis of sequence data. Bioinformatics 28:1647–1649. doi:10.1093/bioinformatics/bts199. 22543367 PMC3371832

[8] Tatusova T, DiCuccio M, Badretdin A, Chetvernin V, Nawrocki EP, Zaslavsky L, Lomsadze A, Pruitt KD, Borodovsky M, Ostell J 2016 NCBI prokaryotic genome annotation pipeline. Nucleic Acids Res 44:6614–6624. doi:10.1093/nar/gkw569. 27342282 PMC5001611

[9] Overbeek R, Olson R, Pusch GD, Olsen GJ, Davis JJ, Disz T, Edwards RA, Gerdes S, Parrello B, Shukla M, Vonstein V, Wattam AR, Xia F, Stevens R 2014 The SEED and the rapid annotation of microbial genomes using subsystems technology (RAST). Nucleic Acids Res 42:D206–D214. doi:10.1093/nar/gkt1226. 24293654 PMC3965101

[10] Zankari E, Hasman H, Cosentino S, Vestergaard M, Rasmussen S, Lund O, Aarestrup FM, Larsen MV 2012 Identification of acquired antimicrobial resistance genes. J Antimicrob Chemother 67:2640–2644. doi:10.1093/jac/dks261. 22782487 PMC3468078

[11] Joensen KG, Scheutz F, Lund O, Hasman H, Kaas RS, Nielsen EM, Aarestrup FM 2014 Real-time whole-genome sequencing for routine typing, surveillance, and outbreak detection of verotoxigenic *Escherichia coli*. J Clin Microbiol 52:1501–1510. doi:10.1128/JCM.03617-13. 24574290 PMC3993690

[12] Arndt D, Grant JR, Marcu A, Sajed T, Pon A, Liang Y, Wishart DS 2016 PHASTER: a better, faster version of the PHAST phage search tool. Nucleic Acids Res 44:W16–W21. doi:10.1093/nar/gkw387. 27141966 PMC4987931

